# Prevalence and Molecular and Antimicrobial Characteristics of *Cronobacter* spp. Isolated From Raw Vegetables in China

**DOI:** 10.3389/fmicb.2018.01149

**Published:** 2018-06-05

**Authors:** Na Ling, Chengsi Li, Jumei Zhang, Qingping Wu, Haiyan Zeng, Wenjing He, Yingwang Ye, Juan Wang, Yu Ding, Moutong Chen, Liang Xue, Qinghua Ye, Weipeng Guo

**Affiliations:** ^1^School of Bioscience and Bioengineering, South China University of Technology, Guangzhou, China; ^2^State Key Laboratory of Applied Microbiology, South China, Guangdong Provincial Key Laboratory of Microbiology Culture Collection and Application, Guangdong Open Laboratory of Applied Microbiology, Guangdong Institute of Microbiology, Guangzhou, China; ^3^College of Food Science, South China Agricultural University, Guangzhou, China; ^4^Department of Food Science & Technology, Jinan University, Guangzhou, China

**Keywords:** *Cronobacter* spp., vegetable, multilocus sequence typing, serotype, antimicrobial sensitivity

## Abstract

*Cronobacter* spp. is a foodborne pathogen that causes life-threatening and invasive diseases, such as necrotizing enterocolitis, meningitis, and sepsis. In this study, we aimed to investigate the prevalence, molecular characteristics and antimicrobial resistance of *Cronobacter* spp. in raw vegetables marketed in China. Based on dietary habits in China, 403 raw vegetables that could be eaten without additional cooking were collected. Of the 403 samples tested, 122 (30.27%) were positive for *Cronobacter* spp., and the contamination levels exceeded 110 most probable number (MPN)/g for 16.39% (20/122) of the samples. Coriander samples had the highest contamination rate of 52.81%, and the MPN values of 19.15% of positive coriander samples exceeded 100 MPN/g. Eleven serotypes were identified among 171 isolates, with *Cronobacter sakazakii* serogroup O1 (41 isolates) being the dominant serotype. Molecular characterization indicated that there was quite high genetic diversity in *Cronobacter* spp., and multilocus sequence typing analyses yielded 106 sequence types (STs), 55 of which were newly identified. Notably, the most prevalent ST (eight isolates) was *C*. *malonaticus* ST60, which appeared in a recent clinical infectious disease study in China. Five *C. sakazakii* ST4, seven *C*. *malonaticus* ST7, and three *C. sakazakii* ST8 confirmed as pathogenic STs in other countries were also detected in this study. Furthermore, all isolates were susceptible to amikacin, amoxicillin-clavulanic, cefepime, ciprofloxacin, and imipenem, but some isolates exhibited a high ratio of resistance to cephalothin (59.65%). In this study, the high contamination rate and the detection of pathogenic and new STs in raw vegetables indicated potential hazards to customers. To the best of our knowledge, this is the first report to provide valuable information on the contamination status of *Cronobacter* spp. in vegetables that can be eaten raw in China.

## Introduction

The genus *Cronobacter* comprises opportunistic pathogens that can cause life-threatening infection in neonates and infants with necrotizing enterocolitis, bacteraemia and meningitis with case fatality rates ranging between 40 and 80% (Sulovska et al., 2017). In addition, infections of *Cronobacter* also occur in older children and adults. In adults, the majority of *Cronobacter* infections are the elderly population, especially those suffering from serious underlying disease or malignancy. In adults, *Cronobacter* can lead to urinary tract infections, septicemia, pneumonia, osteomyelitis, wound infections, and splenic abscesses (Patrick et al., 2014; Alsonosi et al., 2015). The *Cronobacter* genus consists of seven species: *C. sakazakii*, *C. malonaticus*, *C. turicensis*, *C. muytjensii*, *C. dublinensis*, *C. universalis*, and *C. condimenti* (Iversen et al., 2008; Joseph et al., 2012a). Strains belonging to *C*. *sakazakii*, *C*. *malonaticus*, and *C. turicensis* can cause infections in humans (Joseph et al., 2012c). However, the inability of many clinical laboratories to identify *Cronobacter* in a timely manner may allow many infections to go undiscovered (Holy and Forsythe, 2014).

*Cronobacter* spp. have been detected in a wide spectrum of foods, including dairy, cereals, flour, medicinal plants, herbs and spices, rice, meat, fruits, vegetables, and macro fungi, as well as their by-products (Iversen and Forsythe, 2003; Molloy et al., 2009; Cetinkaya et al., 2013; Ye et al., 2014a). The plant environment is considered to be the natural habitat of *Cronobacter* (Schmid et al., 2009). Therefore, non-infant populations could be affected by foods of plant origin contaminated with *Cronobacter* because vegetables are a major part of the normal diet; however, no clear epidemiological link has been established yet between the consumption of raw vegetables and diseases caused by *Cronobacter* spp. Accordingly, more attention should be paid to the food matrices of plant materials due to frequent contamination (Singh et al., 2015). The use of lettuces, corianders, cucumbers, and tomatoes as the main ingredients in ready-to-eat food, such as green salad, is common throughout the world. These four kinds of raw vegetables selected in this study are of great significance, as they are prevalently eaten without cooking, heating or cooling, depending on habitual meal patterns in China. Importantly, outbreaks of *Escherichia coli* and *Salmonella* have been shown to be associated with consumption of lettuce and cucumbers, and these vegetables may represent a major threat to human health (Nygård et al., 2008; Söderström et al., 2008; Zhang et al., 2009; Tuffs, 2011; Taylor et al., 2013; Angelo et al., 2015). However, limited data are available regarding the presence of *Cronobacter* spp. in raw vegetables in China. Therefore, a risk forecast evaluating the contamination status, molecular affiliations, source, and related drug-resistance of *Cronobacter* in vegetables that can be eaten raw is necessary.

Understanding the genetic diversity of *Cronobacter* can contribute to accurate identification at the genus and species levels and may facilitate reliable source tracking of contaminated foods. To date, *Cronobacter* isolates can be further characterized by a range of molecular typing methods (Ye et al., 2014b). *O*-antigen variations are of great significance in epidemiological studies (Blazkova et al., 2015). Based on *O*-antigens, 17 *O*-serogroups of the genus *Cronobacter* have been identified (Mullane et al., 2008; Jarvis et al., 2011, 2013; Sun et al., 2012b). In our previous study, the enterobacterial repetitive intergenic consensus sequence polymerase chain reaction (ERIC-PCR) fingerprint was applied as a serviceable tool for the molecular characterization and identification of *Cronobacter* strains (Ye et al., 2009; Ye et al., 2010). Furthermore, multilocus sequence typing (MLST) based on seven housekeeping genes has proven to be a more robust means of identifying and discriminating species of the *Cronobacter* genus than biotyping, such as 16S rRNA gene sequence analysis, ERIC-PCR and pulsed-field gel electrophoresis (Baldwin et al., 2009; Joseph et al., 2012c). The *Cronobacter* PubMLST database is available online at http://pubmlst.org/cronobacter/. Furthermore, several sequence types (STs) have been shown to be associated with outbreaks (Joseph and Forsythe, 2012). Notably, *C*. *sakazakii* clonal complex 4 was found to be the predominant ST in clinical sources and appeared in the majority of neonatal meningitis cases (Forsythe et al., 2014).

Currently, antibiotic therapy is the clinically preferred and most widespread method for the treatment of *Cronobacter* infection (Depardieu et al., 2007). However, prolonged and extensive use of antibiotics can accelerate bacterial resistance to antimicrobial agents. Recently, *Cronobacter* spp. have been reported to exhibit resistances to amoxicillin-clavulanate, ampicillin, cefazolin, penicillin G, and streptomycin in some isolates (Lee et al., 2012; Xu et al., 2015; Fei et al., 2017). The discovery of the colistin-resistance gene mcr-1 in *Cronobacter* has highlighted the importance of antibiotic resistance in this organism (Liu et al., 2017). Therefore, screening *Cronobacter* isolates from food for antibiotic resistance could be useful in association analysis with clinical isolates and could be of significance to public health and environmental pollution studies.

*Cronobacter* are considered a plant-associated organism and may therefore be highly prevalent in raw vegetables. Therefore, in this study, we aimed to characterize the prevalence of *Cronobacter* spp. in raw vegetables from Chinese markets using phenotyping and genotyping methods in order to determine the genetic relatedness among isolates. We focused on determining the contamination levels, serological types, MLST types, and antibiotic resistance patterns of the isolated strains to characterize the diversity of *Cronobacter* spp. in vegetables from Chinese markets.

## Materials and Methods

### Sampling

**FIGURE 1:**
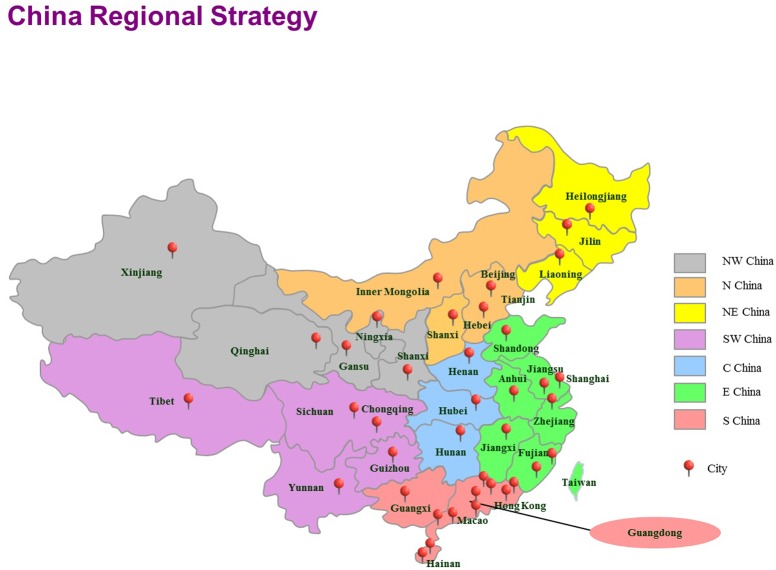
The locations of the sampling sites for this study in China, including 39 cities. Seven geographical divisions based on the Chinese regional strategy are indicated using different colors. E, east; N, north; S, south; NE, northeast; NW, northwest. SW, southwest; C, central.

A total of 403 vegetable samples (lettuce = 87, coriander = 89, tomato = 104, cucumber = 123) were collected from July 2011 to July 2016 in 39 cities in China and tested for the presence of *Cronobacter* isolates (**Table 1**). The sampling sites covered most of the provincial capitals of China, and sites chosen have the certain representation on region. China was divided to seven geographical regions based on the Chinese regional strategy for the topography, climate, humanity, economy and policy of the Chinese government (**Figure 1**). In each city, approximately 10 samples were collected from two supermarkets and two traditional retail markets and supermarkets. Details of sample distribution are shown in Supplementary Table S1. Food samples were transferred under cold conditions (below 4°C) to the laboratory and were immediately subjected to microbiological analysis. Bioexperiments were operated in class II biosafety cabinets in laboratory of BSL2.

**Table 1:** Prevalence and level of *Cronobacter* spp. in different foods.

Food	Prevalence rate (%)	MPN value (MPN/g)		
		MPN < 10 (%)	10 ≤ MPN < 110 (%)	110 ≤ MPN (%)
Lettuce	26/87 (29.89)	17/26 (65.38)	3/26 (11.54)	6/26 (23.08)
Coriander	47/89 (52.81)	30/47 (63.83)	8/47 (17.02)	9/47 (19.15)
Tomato	13/104 (12.50)	11/13 (84.62)	0/13 (0)	2/13 (15.38)
Cucumber	36/123 (29.27)	30/36 (83.33)	3/36 (8.33)	3/36 (8.33)
Total	122/403 (30.27)	88/122 (72.13)	14/122 (11.78)	20/122(16.39)

### Detection of *Cronobacter* spp. Strains

An enrichment method was performed according to the National Food Safety Standard of China for food microbiological examination of *Enterobacter sakazakii* (GB 4789.40-2010, National Standard of the People’s Republic of China, 2010), with minor modifications (Xu et al., 2015). Briefly, 25 g of sample was homogenized for 60 s in stomacher bags (Huankai, Guangzhou, China) with 225 mL of modified lauryl sulfate tryptose broth-vancomycin medium (mLST-Vm), in which vancomycin was added at a final concentration of 10 μg/mL. A nine-tube method was adopted to determine the most probable number (MPN). The nine tubes were divided into three sets of three tubes each. The second and third sets of tubes contained 10 mL of mLST. Three aliquots (10, 1, and 0.1 mL) of the sample homogenate were dispensed into the three sets, representing 1.0, 0.1, and 0.01 g of the original sample, respectively. Nine tubes were incubated at 44°C overnight and were then streaked onto chromogenic medium and incubated at 44°C for 24 h. Presumptive *Cronobacter* spp. presenting green or blue-green colonies were selected for analysis using API 20E diagnostic strips (BioMérieux, Marcy-l’Étoile, France) and species identification was performed by fusA sequencing (Joseph et al., 2012c). MPN was determined on the basis of the number of positive tube(s) in each of the three sets and the MPN table (GB 4789.7-2013, National Standard of the People’s Republic of China, 2013; Xu et al., 2015).

### Molecular Serotyping Identification of *Cronobacter* spp.

The detection of *Cronobacter* spp. serotypes was performed by PCR. Overall, 14 serotypes were identified using protocols and primers according to previously reported *Cronobacter* molecular serotyping schemes, including *C. sakazakii* O:1 to O:7 (Jarvis et al., 2011; Sun et al., 2011, 2012b), *C. turicensis* O:1 to O:3, *C. malonaticus* O:1 and O:2, and *C. dublinensis* O:1 and O:2 (Jarvis et al., 2011, 2013).

### MLST and Sequence Analysis

Multilocus sequence typing was applied to *Cronobacter* spp. molecular typing according to a previous study reported by Joseph et al. (2012b). Seven housekeeping genes were amplified with primers and conditions according to a protocol for *Cronobacter* available at the MLST web database^1^. Comparing the seven loci sequences with this database generated the allele codes and STs of the strains, and the designation of new alleles and STs was verified by the MLST database curator. A neighbor-joining phylogenetic tree was generated based on the seven MLST loci (3,036 bp concatenated length) of the *Cronobacter* spp. strains. This tree was generated using MEGA 5 (version 5.05) with 1000 bootstrap replicates. A minimum spanning tree was constructed in BioNumerics software (Applied Maths, Sint-Martens-Latem, Belgium) according to relationships among MLST alleles.

### Antimicrobial (AM) Susceptibility Test

The susceptibility profile of *Cronobacter* isolates was determined by the standard disk diffusion method using Mueller-Hinton agar (Huankai) following the guidelines of the (Clinical and Laboratory Standards Institute [CLSI], 2006). The following 16 antibiotics (Oxoid, Hampshire, United Kingdom) were classified into nine different groups (**Table 5**) according to the World Health Organization [WHO] (2007): ampicillin (AMP, 10 μg), ampicillin/sulbactam (SAM, 10 μg/10 μg), amoxicillin/clavulanic acid (AMC, 20 μg/10 μg), cefazolin (KZ, 30 μg), cephalothin (KF, 30 μg), cefepime (FEP, 30 μg), ceftriaxone (CRO, 30 μg), gentamicin (CN, 10 μg), tobramycin (TOB, 10 μg), amikacin (AK, 30 μg), chloramphenicol (C, 30 μg), ciprofloxacin (CIP, 5 μg), imipenem (IPM, 10 μg), trimethoprim/sulfamethoxazole (SXT, 1.25 μg/23.75 μg), aztreonam (ATM, 30 μg), and tetracycline (TE, 30 μg). *E. coli* ATCC 25922 was used as a quality control organism.

### Statistical Analysis

Statistical analysis was performed using the SPSS (version 18.0, SPSS, Inc, Chicago, IL, United States) software package. For prevalence data, the chi-square and the Fisher exact test were used for comparison of prevalence rates among the sample sites and prevalence of *Cronobacter* spp. in four vegetables. *P* < 0.05 was considered significant.

## Results

### Prevalence of *Cronobacter* spp. in Non-heat Treated Vegetables

Of the 403 vegetable samples, 122 were confirmed to contain *Cronobacter* spp. (**Table 1**). The prevalence rate of *Cronobacter* varied among the different foods, as follows: 29.89% (26/87) in lettuce, 52.81% (47/89) in coriander, 12.50% (13/104) in tomato, and 29.27% (36/123) in cucumber (**Table 1**). Based on quantitative analysis, 53 of the positive samples (43.44%) were contaminated at a level between 0.3 and 10 MPN/g; contamination levels exceeded 110 MPN/g in 20 samples. Isolates from the same sample belonging to the same ST were considered clonal. Thus, 171 *Cronobacter* spp. isolates were isolated from the 122 positive samples for further study. Of the 171 recovered isolates, the following species were identified: 89 *C. sakazakii* (52.04%), 36 *C. dublinensis* (21.05%), 39 *C. malonaticus* (22.81%), and seven *C. turicensis* isolates (4.10%). The prevalence of *Cronobacter* spp. varied among the seven geographical divisions of China, ranging from 22.45% in Northwest China to 35.21% in South China (**Table 2**). Notably, *C. dublinensis* seemed to be present most often in Central China (*P* < 0.01) (**Table 3**).

**Table 2:** The contamination situation of *Cronobacter* spp. in seven geographical divisions of China.

Region	Positive samples	Negative samples	Total samples	Prevalence rate (%)
S China	50	92	142	35.21%
E China	25	52	77	32.47%
C China	8	21	29	27.59%
N China	11	27	38	28.95%
NE China	7	23	30	23.33%
NW China	11	38	49	22.45%
SW China	10	28	38	26.32%
Total	122	281	403	30.27%

**Table 3:** The distribution of *Cronobacter* species in seven geographical divisions of China.

Region	*C. sakazakii*	*C. malonaticus*	*C. dublinensis*	*C. turicensis*
S China	37/66 (56.06%)	18/66 (27.27 %)	9/66 (13.64%)	2/66 (3.03%)
E China	22/45 (48.89%)	14/45 (31.11%)	7/45 (15.55%)	2/45 (4.44%)
C China	1/13 (7.69%)	1/13 (7.69%)	11/13 (84.61%)	0/13 (0.00%)
N China	10/15 (66.67%)	3/15 (20.00%)	2/15 (13.33%)	0/15 (0.00%)
NE China	5/7 (71.43 %)	0/7 (0.00%)	1/7 (14.28%)	1/7 (14.28%)
NW China	10/13 (76.92%)	1/13 (7.69%)	1/13 (7.69%)	1/13 (7.69%)
SW China	4/12 (33.33%)	2/12 (16.67%)	5/12 (41.67%)	1/12 (8.33%)

### Serogroup Analysis

PCR-based *O*-antigen serotyping techniques were applied to obtain data on the distribution of *O*-antigen serotypes among the 171 *Cronobacter* spp. isolates. The occurrence of *Cronobacter* spp. in the samples is summarized in **Table 4**. The serotypes O:5 and O:6 were not detected in *C. sakazakii* isolates. *C. sakazakii* serotype O:1 was the dominant serotype (41 strains), followed by serotype O:2 (31 strains). Thirty-nine *C*. *malonaticus* strains were classified into two serotypes (O:1 and O:2), with the serotype O:1 covering 20 isolates. Nineteen *C. dublinensis* O:1 and 11 *C. malonaticus* O:2 isolates were identified. Some strains were identified as *C. turicensis* O:1 and O:3.

**Table 4:** Species and serotypes of *Cronobacter* spp. isolates in this study.

Species	Serotype	No. of isolates
*C. sakazakii*	O:1	41
	O:2	31
	O:3	6
	O:4	5
	O:7	6
*C. dublinensis*	O:1	19
	O:2	11
	Uncertain	6
*C. malonaticus*	O:1	20
	O:2	16
	Uncertain	3
*C. turicensis*	O:1	1
	O:3	4
	Uncertain	2
Total		171

*The detection of Cronobacter spp. serotypes was performed by PCR according to Jarvis et al. (2011, 2013) and Sun et al. (2012a; 2012b).*

### Antimicrobial Susceptibility Test

The 171 *Cronobacter* spp. isolates were subjected to 16 antimicrobial susceptibility tests, and the results are shown in **Table 5**. All examined isolates were susceptible to CN, AK, SAM, FEP, CIP, IPM, C, AMC, and ATM. The susceptibility tests revealed that the isolates exhibited relatively high resistance to KF, with resistance and intermediate rates of 59.65 and 38.60%, respectively. Among the remaining tested antibiotics, the next-highest intermediate resistance rate was observed for KZ (28.65 %). Two isolates exhibited resistance to SXT, and three strains showed resistance to TE. However, the two isolates were multidrug-resistant strains that showed resistance to three AMs, i.e., KF, SXT, and TE.

**Table 5:** Antimicrobial resistance profiles of 171 *Cronobacter* spp. isolates.

Antimicrobial group	Antibiotic	Disk code	Antimicrobial class^a^ according to the WHO	No. (%) of *Cronobacter* spp. (*n* = 171)		
				Resistant	Intermediate	Susceptible
Penicillins	Ampicillin	AMP	CI	0 (0)	1 (0.58)	170 (99.42)
	Ampicillin/sulbactam	SAM	CI	0 (0)	0 (0)	171 (100)
	Amoxicillin/clavulanic	AMC	CI	0 (0)	0 (0)	171 (100)
Cephalosporins	Cefepime	FEP	CI	0 (0)	0 (0)	171 (100)
	Ceftriaxone	CRO	CI	0 (0)	4 (2.34)	167 (97.66)
	Cefazolin	KZ	HI	0 (0)	49 (28.65)	122 (71.35)
	Cephalothin	KF	HI	102 (59.65)	66 (38.60)	3 (1.75)
Aminoglycosides	Gentamicin	CN	CI	0 (0)	0 (0)	171 (100)
	Tobramycin	TOB	CI	0 (0)	3 (1.75)	168 (98.25)
	Amikacin	AK	CI	0 (0)	0 (0)	171 (100)
Quinolones	Ciprofloxacin	CIP	CI	0 (0)	0 (0)	171 (100)
Carbapenems	Imipenem	IPM	CI	0 (0)	0 (0)	171 (100)
Sulfonamides	Trimethoprim/sulfamethoxazole	SXT	HI	2 (1.17)	0 (0)	169 (98.83)
Monobactams	Aztreonam	ATM	HI	0 (0)	0 (0)	171 (100)
Amphenicols	Chloramphenicol	C	HI	0 (0)	0 (0)	171 (100)
Tetracyclines	Tetracycline	TE	HI	3 (1.75)	0 (0)	168 (98.25)

*^a^CI, critically important; HI, highly important; I, important.*

### MLST Sequence Analysis of *Cronobacter* spp. Isolates

**FIGURE 2:**
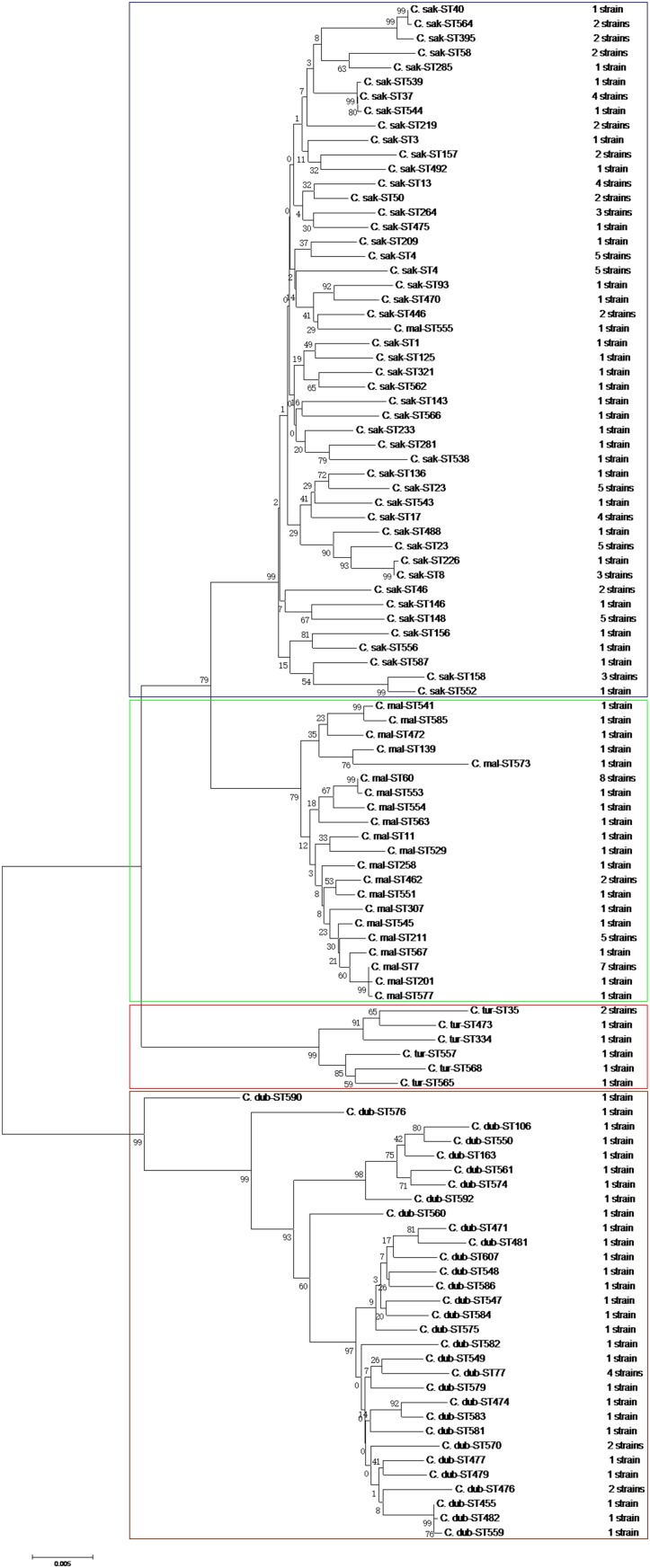
Neighbor-joining phylogenetic tree based on the seven MLST loci (3,036 bp concatenated length) of *Cronobacter* spp. isolates. This tree was generated using MEGA (version 5.05) with 1,000 bootstrap replicates.

**FIGURE 3:**
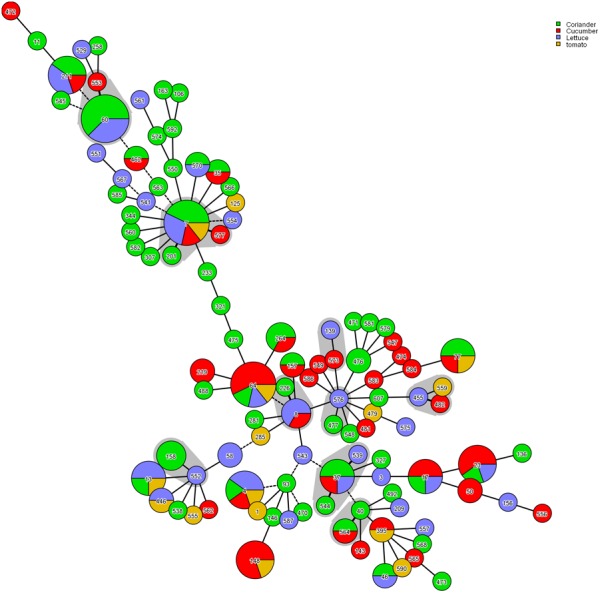
Genetic relationships among the strains isolated in this study. A minimum spanning tree was constructed in BioNumerics software according to relationships among MLST alleles. Strains sharing four or more alleles are surrounding by a gray halo.

The phylogenetic tree generated based on the concatenated sequences of seven loci (**Figure 2**) showed clear clustering across the *Cronobacter* spp.; consequently, the 171 isolates were sorted into four species. BioNumerics (version 7.6) was used to construct a phylogenetic dendrogram to estimate relationships among isolates. Overall, 171 isolates were assigned to 106 STs, of which 55 were novel (Supplementary Table S2). Among these 106 STs, 79 of the 106 STs were represented only by one strain, and the remaining 27 STs covered 2–8 isolates. ST60 was the dominant ST (*n* = 8), followed by ST7 and ST64, which each included seven strains. ST4, ST23, ST148, and ST211, which were associated with clinical isolates, included five isolates. Furthermore, ST4, ST64, and ST7 were found in all four vegetables; ST17, ST23, ST37, and ST211 were found in coriander, lettuce, and cucumber; ST77 was found in coriander, cucumber, and tomato; ST13 was found in coriander, lettuce, and tomato; and ST60 was only detected in coriander and cucumber (**Figure 3**).

## Discussion

*Cronobacter* spp. are responsible for fatal infections caused by ingestion of contaminated infant formula (Van Acker et al., 2001; Stoll et al., 2004; Li et al., 2016). Additionally, *Cronobacter* isolates may be present in a diverse range of environments and in foods that are commonly consumed. Because China is a large agricultural country with relatively abundant resources, considerable attention should be paid to vegetable safety issues related to microorganisms. To date, limited data are available regarding *Cronobacter* spp. present in raw vegetables.

In this study, owing to common Chinese dietary habits, 403 raw vegetables that could be eaten without additional cooking were examined. Overall, the prevalence of *Cronobacter* spp. in vegetables was determined to be 30.27%; contamination levels of *Cronobacter* spp. exceeded 110 MPN/g in 20 samples. Notably, coriander samples had the highest contamination rate of 52.81% (*P* < 0.01), and the MPN value of 19.15% of positive coriander samples exceeded 100 MPN/g. However, the methodology used in this study has not been standardized for vegetables, and it was developed and standardized for detecting *Cronobacter* spp. from powdered infant formula; thus, the isolation rates may have been underestimated. Several previous studies have reported the occurrence of *Cronobacter* strains in vegetables. For example, Chon et al. (2012) and Lee et al. (2012) isolated *Cronobacter* spp. in 14.8% and 30% of vegetables in Korea, respectively. In addition, a study on the prevalence of this bacteria in plant-based food and related environments was undertaken in the Czech Republic and detected *Cronobacter* spp. in 8.0% of samples (Vojkovska et al., 2016). Recently, 15 of 102 vegetable samples were found to be positive in the middle-east coastline of China (Chen et al., 2016). Furthermore, in our previous study, samples of cold vegetables in sauce were contaminated with *Cronobacter* spp. at a particularly high rate of 41.9% (Xu et al., 2015). This great variability observed in different studies is probably due to the number and species of samples analyzed and cross-contamination resulting from open vegetable display areas. In addition, the prevalence of *Cronobacter* spp. varied in seven geographical divisions of China, ranging from 22.45% in Northwest China to 36.11% in South China. Surprisingly, the presence of *C. dublinensis* in Central China was significantly higher than those in other regions (*P* < 0.01); however, the exact reason for this phenomenon is still unknown. Because the sample size was not large enough, continuous surveillance of *Cronobacter* in China for reliable source tracking and determination of the contamination status is necessary. Additionally, no standards for limitation of *Cronobacter* spp. in vegetables have been established in China. Lettuce and cucumbers caused the life-threatening infections owing to *E*. *coli* and *Salmonella* contamination. Cases of human *Cronobacter* infection have not been linked to its presence in raw vegetables, however, raw vegetables may potentially disperse *Cronobacter* and may contribute to human infections. Particularly immune-compromised individuals, the elderly and hospitalized patients may be at risk when eating raw vegetables without heating.

In this study, 171 *Cronobacter* spp. isolates were isolated from 122 positive samples. Fourteen *Cronobacter* serogroups have been identified. Among this collection of isolates, representatives of all but two *O*-antigen serotypes (O:5 and O:6) were recognized, consistently with several previous studies (Xu et al., 2014, 2015). *C. sakazakii* serotype O:1 was the dominant serotype, followed by serotype O:2. PCR-based *O*-antigen serotyping was reliable for characterizing isolates of *E. coli* and *Listeria monocytogenes* from clinical, food, and environmental samples (Singh and Manning, 2014; Chen et al., 2015). However, until recently there were only 17 defined serotypes across the whole *Cronobacter* genus (Mullane et al., 2008; Jarvis et al., 2011, 2013; Sun et al., 2012b), and the identification of unknown serotypes in this study indicated that an integrated standardized molecular serotyping protocol should be developed. Furthermore, there was also a contradiction in the specificity of the molecular serotyping classification presented by Jarvis et al. (2011), in which *C. sakazakii* O:3 and *C. muytjensii* O:1 were identified to be the same serogroup. Thus, further data on the distribution of serotypes (e.g., among clinical isolates) are needed to determine whether molecular serotyping is a useful alternative for epidemiological surveillance of *Cronobacter*.

Multilocus sequence typing is based on the sequences of gene fragments from a number of different housekeeping loci, and has also been used successfully to subtype *Cronobacter* isolates and to study the evolution and population genetics of various *Cronobacter* spp. (Joseph and Forsythe, 2012). MLST revealed 106 STs from the 171 *Cronobacter* isolates, of which 15 STs (ST1, ST4, ST7, ST8, ST13, ST23, ST40, ST60, ST64, ST93, ST158, ST233, ST264, ST281, and ST307) were associated with clinical isolates in different countries according to the PubMLST database (see footnote 1). ST4, ST7, ST8, and ST60 are particularly noteworthy. *Cronobacter* ST60 was the dominant ST in the current study. Unfortunately, one case of *Cronobacter* ST60 infection in an infant was detected at Wuhan Women and Children Medical Care Center Hospital (Wuhan City, China), and the pathogenesis mechanism of ST60 is still unclear (Cui et al., 2017). Therefore, we should increase our vigilance toward ST60 in raw vegetable as these can be a potential source of disease in children and the rest of the population; however, there is still not clear evidence of an epidemiological link between the consumption of raw vegetables and infection caused by *Cronobacter*. ST4 is the most dominant ST in clonal complex 4, which is regarded as a genetic signature for neonatal meningitis. To date, it is unclear why *C. sakazakii* clonal complex 4 predominates neonatal meningitis cases (Joseph and Forsythe, 2011; Hariri et al., 2013; Forsythe et al., 2014). Furthermore, seven *C. malonaticus* ST7 strains were also detected in this study. Considering that *C. malonaticus* ST7 is more often associated with adult infections than neonatal infections (Joseph and Forsythe, 2011; Alsonosi et al., 2015), and that ST7 is detected in uncooked vegetables served in ready-to-eat foods or salads, which adults eat more often than do neonates, raw vegetables may be a potential route of transmission and potential reservoirs for these infections. Three strains belonging to ST8 were also isolated. To date, although no ST8 strains have been shown to be associated with severe diseases, such as meningitis, but with diarrhea (Joseph and Forsythe, 2012), further studies of ST8 isolates are needed. Notably, most of these isolates harboring pathogenic STs tended to be located in South China. Surprisingly, up to 55 new STs were detected, indicating that further studies of the genetic populations of the *Cronobacter* genus are necessary to identify more STs, which could assist in risk identification and epidemiological studies of this pathogen.

According to molecular characterizations, there was high genetic diversity among the *Cronobacter* spp. in raw vegetables. MLST has high discriminatory power capable of showing genetic diversity among *Cronobacter* spp. isolates and can distinguish among the seven species using patterns of molecular markers. Furthermore, our study showed that the MLST patterns were generally associated with serotypes and provided a reliable prediction of the *Cronobacter* serovars. For instance, all *C. malonaticus* ST60 strains corresponded with the O:1 serotype. The association was not exclusive, however, as the seven isolated *C. malonaticus* ST7 strains corresponded with the O:2 serotype. The *Cronobacter* pathovar results agreed well with previous research (Ogrodzki and Forsythe, 2015). Continuous and comprehensive investigations should be carried out to improve our understanding of the genetic evolution of *Cronobacter* spp.

Because contaminated vegetables are considered transmission sources for human clinical isolates, the antimicrobial profiles of the isolates from raw vegetables were examined. All isolates were susceptible to AK, AMC, FEP, CIP, and IPM. However, some isolates exhibited high resistance to KF and intermediate resistance to KF and KZ, which was consistent with other studies (Molloy et al., 2009; Chon et al., 2012). In addition, the chloramphenicol and gentamicin resistance rates were significantly different than those of *Cronobacter* spp. isolated from Chinese ready-to-eat foods in our laboratory (Xu et al., 2015) and various foods studied by Lee et al. (2012). The heterogeneity between vegetables and ready-to-eat foods suggested that the survival environment and source were vital for drug resistance. Notably, two isolates were multidrug-resistant strains (showing resistance to KF, SXT, and TE) in this study. These two multidrug-resistant isolates belonged to epidemiologically new STs, suggesting that these multidrug-resistant strains may have acquired resistance by vertical and horizontal gene transfer or from other microorganisms. Therefore, more attention is required while monitoring variations in the resistance patterns toward SXT and TE. In other studies, extended-spectrum β-lactamase-related genes (Girlich et al., 2001) and the colistin-resistance gene mcr-1 (Liu et al., 2017) were found in *Cronobacter*. AMP/CN or AMP/C usually serves as traditional therapy for *Cronobacter* spp. (Lai, 2001); therefore, intermediate resistance to AMP in *Cronobacter* isolates from raw vegetables may interfere with therapeutic approaches. Furthermore, some isolates exhibited intermediate resistance to CRO, TOB, and AMP. This could lead to the emergence of resistant strains under certain circumstances (Ruiz-Bolivar et al., 2011). Therefore, further studies are needed to determine the route of bacterial resistance and to moderate the use of drugs in order to prevent the emergence of antimicrobial resistance.

## Conclusion

Overall, the findings of this study demonstrated a high contamination rate of *Cronobacter* in raw vegetable samples in China and suggested that raw vegetables may be potential carrier and transmission sources of *Cronobacter*. Based on MLST analyses, the pathogenic STs ST60, ST4, ST7, and ST8 were detected in raw vegetables; these STs would pose potential risks to both children and adults. In addition, all isolates were susceptible to AK, AMC, FEP, CIP, and IPM. However, strains showing resistance to KF and intermediate resistance to KF and KZ were found at a high ratio in raw vegetables. In this study, the high contamination rate and detection of pathogenic and new STs in raw vegetables indicated potential hazards to customers. To date, no standards for limitation of *Cronobacter* spp. in raw vegetables have been established in China. Thus, these findings highlight the need for dynamic screening related to transmission routes and the risk of food contamination of vegetables in China. However, the sample size was not large enough in this study, continuous surveillance of *Cronobacter* in China for reliable source tracking and determination of the contamination status is necessary.

## Author Contributions

NL, CL, and JZ contributed to the manuscript equally, done the article experiments and wrote the article together. QW gave the idea and experiments support. NL, JZ, and QW conceived the project. NL, CL, HZ, WH, YY, JW, YD, MC, WG, LX, and QY performed the experiments. JZ and QW supervised the project. NL and CL analyzed the data. NL wrote the article.

## Conflict of Interest Statement

The authors declare that the research was conducted in the absence of any commercial or financial relationships that could be construed as a potential conflict of interest.

## Footnotes

1 http://pubmlst.org/cronobacter/
