# Supplementary material for: Prevalence and Molecular and Antimicrobial Characteristics of Cronobacter spp. Isolated From Raw Vegetables in China
Source: Front Microbiol. 2018 Jun 5;9:1149. doi: 10.3389/fmicb.2018.01149 (PMC5996200; doi:10.3389/fmicb.2018.01149)
Supplement: Supplementary file 1 [file Data_Sheet_1.docx]

**Prevalence and Molecular and Antimicrobial Characteristics of *Cronobacter* spp. Isolated from Raw Vegetables** **in China**

**Na Ling ^1,^** **^2, a^,** **Chengsi Li ^2, a^, Jumei Zhang ^2, a^, Qingping Wu ^2^*, Haiyan Zeng ^2^, Wenjing He ^2^, Yingwang Ye ^2^, Juan Wang ^2^, Yu Ding ^2^, Moutong Chen ^2^, Liang Xue ^2^, Qinghua Ye ^2^, Weipeng Guo ^2^**

***Correspondence:**Qingping Wu
wuqp203@163.com, wuqp@gdim.cn

**Supplementary Information contains:**

Supplementary Table S1 and Table S2

**Table S1.** Details of sample distribution in China

| Region | Province/ Municipality/ Autonomous region | City | No. of sample |
| --- | --- | --- | --- |
| S China | Guangdong | Guangzhou | 45 |
|  |  | Shenzhen | 8 |
|  |  | Shantou | 9 |
|  |  | Shaoguan | 9 |
|  |  | Zhanjiang | 8 |
|  |  | Heyuan | 9 |
|  | Hainan | Haikou | 9 |
|  |  | Sanya | 8 |
|  | Guangxi | Nanning | 8 |
|  |  | Beihai | 9 |
|  | Hong Kong | Hong Kong | 10 |
|  | Macao | Macao | 10 |
| E China | Shandong | Jinan | 10 |
|  | Jiangsu | Nanjin | 10 |
|  | Shanghai | Shanghai | 10 |
|  | Anhui | Hefei | 9 |
|  | Zhejiang | Hangzhou | 10 |
|  | Jiangxi | Nanchang | 10 |
|  | Fujian | Fuzhou | 9 |
|  | Fujian | Xiamen | 9 |
| C China | Henan | Zhenzhou | 10 |
|  | Hubei | Wuhan | 9 |
|  | Hunan | Changsha | 10 |
| N China | Inner Mongolia | Huhehaote | 10 |
|  | Shanxi | Xian | 9 |
|  | Beijing | Beijing | 9 |
|  | Hebei | Shijiazhuang | 10 |
| NE China | Heilongjiang | Haerbing | 10 |
|  | Jilin | Changchun | 10 |
|  | Liaoning | Shenyang | 10 |
| NW China | Xinjiang | Wulumuqi | 10 |
|  | Qinghai | Xining | 10 |
|  | Gansu | Lanzhou | 10 |
|  | Ningxia | Yinchuan | 10 |
|  | Shanxi | Taiyuan | 9 |
| SW China | Tibet | Lasa | 10 |
|  | Sichuan | Chengdu | 9 |
|  | Yunnan | Kunming | 9 |
|  | Guizhou | Guiyang | 10 |
| Total |  |  | 403 |

**Table S2.** Information of MLST alleles of new STs.

| ID | Strain | Species | ST | atpD | fusA | glnS | gltB | gyrB | infB | pps |
| --- | --- | --- | --- | --- | --- | --- | --- | --- | --- | --- |
| 1873 | Cro1014W | *C. dublinensis* | 582 | 40 | 48 | 216 | 152 | 149 | 149 | 302 |
| 1810 | Cro1046A1 | *C. dublinensis* | 549 | 160 | 20 | 213 | 232 | 205 | 202 | 277 |
| 1811 | Cro1364W | *C. dublinensis* | 550 | 112 | 41 | 221 | 233 | 206 | 203 | 278 |
| 1812 | Cro1413A2 | *C. malonaticus* | 551 | 10 | 13 | 67 | 234 | 131 | 124 | 174 |
| 1813 | Cro1413B2 | *C. sakazakii* | 552 | 15 | 17 | 193 | 36 | 86 | 90 | 106 |
| 1874 | Cro1441W | *C. dublinensis* | 584 | 70 | 43 | 228 | 252 | 221 | 215 | 303 |
| 1800 | Cro154 | *C. sakazakii* | 539 | 44 | 15 | 3 | 235 | 5 | 38 | 59 |
| 1847 | Cro1541A1-1 | *C. turicensis* | 557 | 6 | 49 | 39 | 52 | 52 | 59 | 72 |
| 1814 | Cro1541W2 | *C. malonaticus* | 567 | 10 | 162 | 67 | 7 | 77 | 204 | 279 |
| 1848 | Cro1565W | *C. malonaticus* | 573 | 165 | 13 | 199 | 74 | 72 | 41 | 88 |
| 1815 | Cro1591W | *C. malonaticus* | 553 | 12 | 160 | 8 | 8 | 10 | 16 | 43 |
| 1816 | Cro1764W1 | *C. malonaticus* | 554 | 10 | 7 | 18 | 7 | 207 | 205 | 43 |
| 1817 | Cro1790W | *C. sakazakii* | 555 | 55 | 17 | 194 | 236 | 15 | 56 | 125 |
| 1849 | Cro1813A3 | *C. dublinensis* | 576 | 151 | 20 | 71 | 90 | 8 | 41 | 256 |
| 1818 | Cro1814W | *C. turicensis* | 568 | 60 | 65 | 39 | 86 | 76 | 80 | 94 |
| 1819 | Cro1841W2 | *C. sakazakii* | 556 | 71 | 1 | 13 | 18 | 208 | 56 | 78 |
| 1832 | Cro1914W2 | *C. sakazakii* | 566 | 159 | 167 | 195 | 238 | 209 | 206 | 280 |
| 1867 | Cro197 | *C. dublinensis* | 575 | 161 | 20 | 217 | 245 | 119 | 212 | 296 |
| 1850 | Cro2031W | *C. malonaticus* | 585 | 166 | 7 | 177 | 7 | 17 | 141 | 304 |
| 1822 | Cro2090B3 | *C. dublinensis* | 559 | 62 | 23 | 196 | 85 | 79 | 83 | 281 |
| 1823 | Cro2114W | *C. dublinensis* | 560 | 113 | 125 | 229 | 164 | 210 | 207 | 214 |
| 1824 | Cro2163B2 | *C. dublinensis* | 561 | 94 | 100 | 113 | 133 | 211 | 130 | 282 |
| 1825 | Cro2215A1 | *C. sakazakii* | 562 | 15 | 17 | 9 | 239 | 212 | 208 | 283 |
| 1852 | Cro2241A3 | *C. dublinensis* | 586 | 122 | 20 | 230 | 253 | 82 | 41 | 305 |
| 1827 | Cro2264C3 | *C. sakazakii* | 564 | 3 | 15 | 28 | 22 | 5 | 38 | 284 |
| 1826 | Cro2264W | *C. malonaticus* | 563 | 158 | 7 | 12 | 7 | 9 | 35 | 166 |
| 1828 | Cro2291A1 | *C. sakazakii* | 564 | 3 | 15 | 28 | 22 | 5 | 38 | 284 |
| 1868 | Cro244 | *C. dublinensis* | 579 | 162 | 165 | 225 | 246 | 164 | 213 | 297 |
| 1860 | Cro2691W | *C. dublinensis* | 583 | 30 | 20 | 231 | 254 | 154 | 27 | 303 |
| 1829 | Cro2764W2 | *C. turicensis* | 565 | 115 | 88 | 39 | 244 | 213 | 209 | 285 |
| 1853 | Cro2790W | *C. dublinensis* | 590 | 157 | 63 | 39 | 123 | 226 | 212 | 306 |
| 1865 | Cro280B | *C. dublinensis* | 476 | 58 | 20 | 214 | 213 | 164 | 158 | 255 |
| 1675 | Cro2814C3 | *C. dublinensis* | 471 | 58 | 20 | 99 | 89 | 83 | 43 | 117 |
| 1676 | Cro2841W | *C. malonaticus* | 472 | 88 | 143 | 17 | 204 | 77 | 156 | 90 |
| 1677 | Cro2864C1 | *C. turicensis* | 473 | 115 | 88 | 181 | 210 | 192 | 157 | 203 |
| 1679 | Cro2915W | *C. dublinensis* | 474 | 30 | 20 | 33 | 211 | 128 | 129 | 294 |
| 1680 | Cro3114W | *C. sakazakii* | 475 | 16 | 8 | 9 | 32 | 21 | 113 | 253 |
| 1799 | Cro32 | *C. sakazakii* | 538 | 156 | 163 | 19 | 15 | 18 | 90 | 286 |
| 1682 | Cro3214W | *C. dublinensis* | 570 | 150 | 144 | 179 | 212 | 193 | 194 | 254 |
| 1683 | Cro3314A1 | *C. dublinensis* | 476 | 58 | 20 | 214 | 213 | 164 | 158 | 255 |
| 1684 | Cro3314A3-1 | *C. dublinensis* | 477 | 151 | 20 | 224 | 90 | 194 | 41 | 256 |
| 1687 | Cro3540W | *C. dublinensis* | 479 | 152 | 20 | 71 | 91 | 195 | 212 | 36 |
| 1689 | Cro4191A2 | *C. dublinensis* | 481 | 153 | 20 | 180 | 214 | 34 | 222 | 258 |
| 1690 | Cro4191B1-1 | *C. dublinensis* | 482 | 62 | 23 | 122 | 85 | 79 | 83 | 259 |
| 1866 | Cro50 | *C. malonaticus* | 577 | 10 | 7 | 6 | 7 | 9 | 14 | 295 |
| 1802 | Cro595C2 | *C. malonaticus* | 541 | 10 | 7 | 67 | 7 | 17 | 141 | 287 |
| 1804 | Cro663A2 | *C. sakazakii* | 543 | 15 | 8 | 3 | 5 | 5 | 24 | 288 |
| 1805 | Cro664B1-2 | *C. sakazakii* | 544 | 44 | 15 | 3 | 5 | 5 | 38 | 289 |
| 1862 | Cro795W1 | *C. sakazakii* | 587 | 16 | 36 | 3 | 249 | 58 | 36 | 4 |
| 1806 | Cro864B2-3 | *C. malonaticus* | 545 | 10 | 7 | 17 | 8 | 214 | 16 | 290 |
| 1795 | Cro913W | *C. malonaticus* | 529 | 64 | 13 | 8 | 75 | 17 | 16 | 274 |
| 1871 | Cro914C1 | *C. dublinensis* | 607 | 163 | 23 | 114 | 247 | 235 | 41 | 300 |
| 1808 | Cro946B2 | *C. dublinensis* | 547 | 58 | 20 | 226 | 240 | 215 | 210 | 291 |
| 1855 | Cro964A2 | *C. dublinensis* | 592 | 37 | 41 | 26 | 27 | 219 | 32 | 163 |
| 1872 | Cro964W | *C. dublinensis* | 581 | 58 | 20 | 191 | 250 | 203 | 199 | 301 |
| 1809 | Cro981C1 | *C. dublinensis* | 548 | 157 | 20 | 215 | 241 | 44 | 41 | 293 |
| 1858 | Cro981C2 | *C. dublinensis* | 574 | 112 | 41 | 198 | 251 | 211 | 154 | 109 |
| 1857 | Cro981W | *C. dublinensis* | 570 | 150 | 144 | 179 | 212 | 193 | 194 | 254 |
